# Elucidating the rate-limiting step of CO_2_ electroreduction on metal phthalocyanines

**DOI:** 10.1038/s41467-026-70445-9

**Published:** 2026-03-10

**Authors:** Zhuanghe Ren, Kaige Shi, Zhen Meng, Thomas Egan, Talat S. Rahman, Xiaofeng Feng

**Affiliations:** 1 Department of Physics, University of Central Florida, Orlando, FL USA; 2 Department of Chemistry, University of Central Florida, Orlando, FL USA; 3 Renewable Energy and Chemical Transformations (REACT) Cluster, University of Central Florida, Orlando, FL USA; 4 Department of Materials Science and Engineering, University of Central Florida, Orlando, FL USA

**Keywords:** Electrocatalysis, Electrocatalysis, Electrocatalysis

## Abstract

Immobilized molecular catalysts, especially metal phthalocyanines, have garnered substantial interest for the electrochemical CO_2_ reduction reaction (CO_2_RR) due to their well-defined active sites and promising performance. Yet, the reaction mechanism, particularly the rate-limiting step, remains debated. Here, using electrochemical analysis and kinetic isotope effect measurements, we identify the rate-limiting step of CO_2_RR to CO on immobilized metal phthalocyanines, with Au as a reference. Notably, cobalt phthalocyanine (CoPc) exhibits dispersion-dependent kinetics: protonation of adsorbed *CO_2_ is rate-limiting on molecularly dispersed CoPc supported on carbon nanotubes (CoPc/CNTs), whereas CO_2_ adsorption becomes rate-limiting on aggregated CoPc due to a weakened interfacial electric field at the Co active sites. This mechanistic distinction further elucidates the role of electrolyte anions: HCO_3_^−^, largely a spectator on Au, promotes CO_2_RR on CoPc/CNTs by serving as a proton donor in the rate-limiting protonation step. These findings provide mechanistic insights into CO_2_RR on metal phthalocyanines and guide the rational design of molecular electrocatalysts.

## Abstract (web summary)

Metal phthalocyanines are promising molecular electrocatalysts for CO_2_ conversion, but the rate-limiting step remains unresolved. Here, the authors show that it depends on catalyst dispersion, shifting from protonation in molecularly dispersed catalysts to CO_2_ adsorption in molecular aggregates.

## Introduction

Electrochemical CO_2_ reduction reaction (CO_2_RR) powered by renewable electricity offers a promising pathway toward carbon neutrality by transforming abundant carbon emissions into value-added chemicals^1–3^. To achieve the techno-economic viability of this process, it is essential to develop highly active and selective catalysts^4^. Metallic materials have been widely employed to catalyze CO_2_RR, resulting in a range of products including CO^5–8^, formate^9,10^, and multi-carbon hydrocarbons and oxygenates such as ethylene, ethanol, and propanol^11–14^. Although substantial progress has been made in improving the CO_2_RR performance of metal catalysts, the inherent multiplicity of active sites hinders a clear mechanistic understanding and rational catalyst design. Another challenge for metal catalysts is to achieve high CO_2_RR selectivity due to the competing hydrogen evolution reaction (HER)^15,16^, especially in acidic media. In contrast, molecular catalysts with well-defined metal centers have demonstrated notable activity for CO_2_RR and are relatively inert toward HER, providing a promising alternative to metal catalysts^17^.

Molecular catalysts with a variety of transition metal centers and macrocyclic structures have been investigated for CO_2_RR^18–21^. They are typically immobilized on carbon substrates and exhibit promising catalytic performance for the CO_2_RR to CO. For instance, Faradaic efficiencies for CO production exceeding 90% at relatively low overpotentials have been reported for the CO_2_RR on cobalt phthalocyanines (CoPc)^22–24^, cobalt porphyrins^25,26^, cobalt quaterpyridine complexes^27^, nickel phthalocyanines (NiPc)^28^, and functionalized iron porphyrins^29,30^. Among these molecules, metal phthalocyanines have been the most extensively studied due to their facile availability, high CO_2_RR activity, and capability of producing more reduced products such as methanol^31–35^. Despite the advances in catalytic performance, the reaction mechanism, particularly the rate-limiting step, remains elusive for CO_2_RR on metal phthalocyanines^36–39^.

**Fig. 1:**
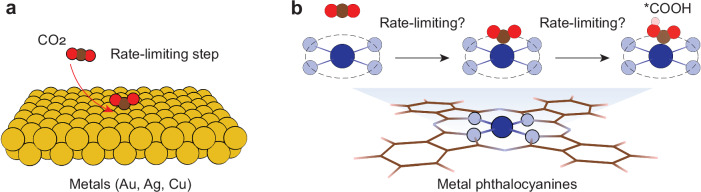
Proposed rate-limiting steps of the CO_2_RR on different catalysts. **a** CO_2_ adsorption is identified as the rate-limiting step for the CO_2_RR to CO on typical metal catalysts. **b** For metal phthalocyanines, both CO_2_ adsorption and the subsequent protonation of adsorbed *CO_2_ have been proposed as rate-limiting steps.

For CO_2_RR to CO on metal catalysts, CO_2_ adsorption is generally considered to be the rate-limiting step, as schematically illustrated in Fig. 1a. However, on metal phthalocyanines such as CoPc, both CO_2_ adsorption and subsequent protonation of adsorbed *CO_2_ have been proposed as rate-limiting steps (Fig. 1b). McCrory and co-workers evaluated the kinetic isotope effect (KIE) for the CO_2_RR on CoPc immobilized on a graphite disk electrode^37^. They found that the CO_2_RR activity remained nearly identical in aprotic and deuterated phosphate electrolytes, indicative of a rate-limiting step without proton involvement, and consequently, identified the CO_2_ adsorption step as rate-determining. A similar result was reported for CO_2_RR on CoPc supported on carbon nanotubes (CNTs)^32^. In contrast, Chan and co-workers suggested that CO_2_RR on CNT-supported CoPc is limited by the protonation of adsorbed *CO_2_ through a combination of computations and pH-dependent measurements^38^. This conclusion was supported by the work of Feng et al., in which the KIE studies showed a significantly higher CO_2_RR activity in H_2_SO_4_ compared to that in D_2_SO_4_, indicating that the protonation of *CO_2_ is the rate-limiting step^39^. Given these controversial results, it is crucial to elucidate the rate-limiting step of CO_2_RR on metal phthalocyanines.

In this work, we investigate and elucidate the rate-limiting step of the CO_2_RR to CO on metal phthalocyanines, with Au catalyst as a reference. By combining electrochemical measurements and KIE analysis, we show that the CO_2_RR on molecularly dispersed CoPc and NiPc supported on CNTs (CoPc/CNTs and NiPc/CNTs) is limited by the protonation of adsorbed *CO_2_. Using CoPc as a model system, we further demonstrate that the rate-limiting step of CO_2_RR depends on the catalyst’s dispersion state, shifting to CO_2_ adsorption on aggregated CoPc due to a weakened interfacial electric field at the Co active sites. Considering the rate-limiting protonation step on CoPc/CNTs, we further examine the often-overlooked role of electrolyte anions in CO_2_RR and reveal a more pronounced effect of HCO_3_^−^ relative to Na^+^, in contrast to observations on typical metal catalysts. The promotional effect of HCO_3_^−^ is attributed to its role as a proton donor in the protonation of *CO_2_. Our work clarifies the reaction mechanism and rate-limiting step of the CO_2_RR on metal phthalocyanines, thereby laying the groundwork for the rational design of efficient molecular electrocatalysts.

## Results

### Characterization of CoPc/CNTs and NiPc/CNTs catalysts

**Fig. 2:**
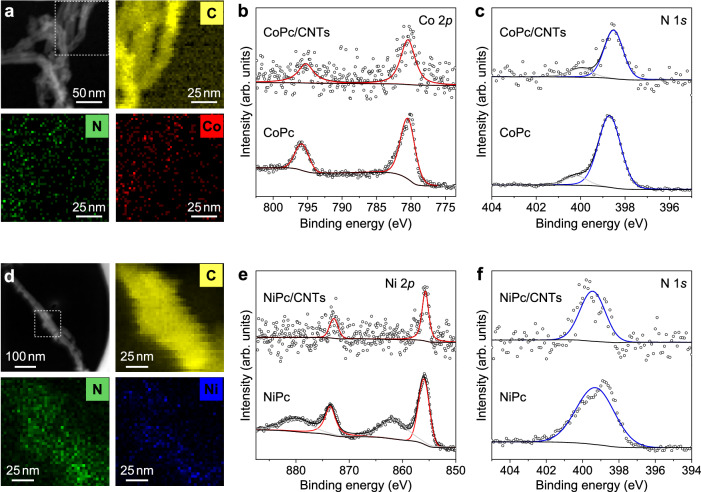
Characterization of the CoPc/CNTs and NiPc/CNTs catalysts. STEM image and corresponding EDS mapping (**a**), and high-resolution XPS spectra of Co 2*p* (**b**) and N 1*s* (**c**) of the CoPc/CNTs sample. STEM image and corresponding EDS mapping (**d**), and high-resolution XPS spectra of Ni 2*p* (**e**) and N 1*s* (**f**) of the NiPc/CNTs sample. Source data are provided as a Source data file.

Molecular catalysts are typically integrated into electrodes by pre-immobilization or direct deposition onto carbon nanomaterials to achieve molecular dispersion^18^. When anchored onto CNT supports, the molecular catalysts can exhibit high activity and selectivity for CO_2_RR, which is attributed to the increased exposure of active sites and enhanced electron transfer^18^. Therefore, we prepared CoPc/CNTs and NiPc/CNTs samples using a well-established anchoring strategy (see Methods for details)^31^. To contextualize our findings within the broader CO_2_RR field, we selected Au as a benchmark catalyst. Therefore, Au nanoparticles supported on carbon black (Au/C) were also prepared to serve as a reference due to its well-understood catalytic mechanism for CO_2_RR. The three samples were characterized for their morphology, structure, and chemical state. As shown in Fig. 2a, d, scanning transmission electron microscopy (STEM) images and corresponding energy-dispersive X-ray spectroscopy (EDS) mapping indicate a homogeneous dispersion of CoPc and NiPc on CNTs in the CoPc/CNTs and NiPc/CNTs samples, respectively. High-resolution X-ray photoelectron spectra (XPS) of Co 2*p* and N 1*s* regions (Fig. 2b, c) as well as Ni 2*p* and N 1*s* regions (Fig. 2e, f) confirm the presence of CoPc and NiPc molecules in the samples. Moreover, both scanning electron microscopy (SEM) and transmission electron microscopy (TEM) images indicate that the morphology of CoPc/CNTs and NiPc/CNTs resembles that of the pristine CNTs, showing no visible molecular aggregates (Supplementary Figs. 1 and 2). Their X-ray diffraction (XRD) patterns further support the absence of crystalline aggregates of CoPc and NiPc, as their characteristic diffraction peaks were not observed (Supplementary Figs. 1 and 2). Collectively, these results confirm a molecular dispersion state of CoPc and NiPc on CNTs. For the Au/C nanopowder, TEM, XRD, and XPS characterizations indicate an average particle size of around 5 nm and a metallic state of the Au nanoparticles, as shown in Supplementary Fig. 3. Each of the three samples was deposited onto an AvCarb GDS2230 carbon substrate to form the corresponding electrode.

### Identification of the rate-limiting step

To identify the rate-limiting step of CO_2_RR, we employ the KIE method, as it can determine proton involvement in the key step, thereby distinguishing between the steps of CO_2_ adsorption and protonation of adsorbed *CO_2_. The magnitude of KIE is defined by *j*_H_/*j*_D_, where *j*_H_ is the partial current density for a specific product in a protic electrolyte and *j*_D_ is that in a deuterated electrolyte^37^. If the rate-limiting step is a protonation process, a KIE value >1 will be observed due to the sluggish dissociation of deuterated water and deuteron transfer process. In contrast, a rate-limiting step without proton involvement should result in a KIE value of around 1. To evaluate the KIE, CO_2_RR bulk electrolysis was performed in a two-compartment electrochemical cell (H-cell, Supplementary Fig. 4) with 1 M NaHCO_3_ in H_2_O and 1 M NaDCO_3_ in D_2_O electrolytes, respectively. All potentials were 100% *iR*-compensated using the current-interrupt method (unless otherwise specified) and are reported on the reversible hydrogen electrode (RHE) scale throughout this work. Gas-phase products, including CO and H_2_, were quantified using gas chromatography (GC), and liquid-phase products were analyzed by ^1^H nuclear magnetic resonance (NMR) spectroscopy.

**Fig. 3:**
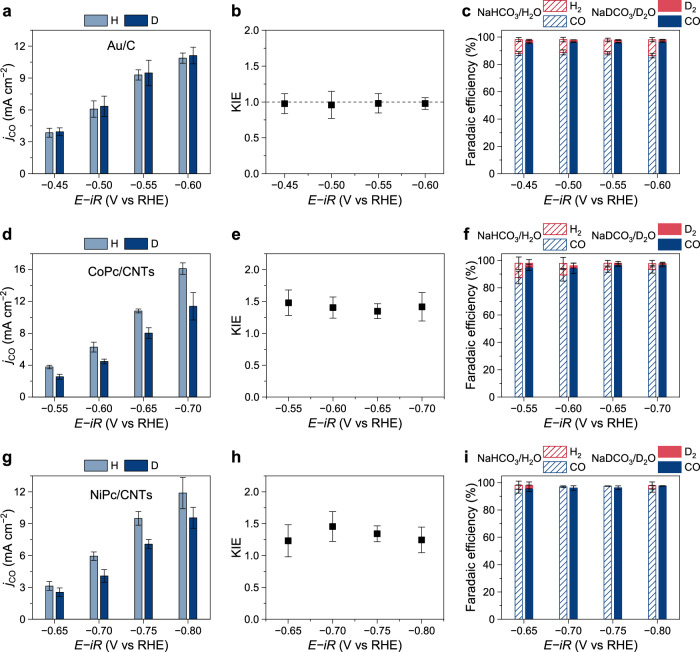
KIE analysis of CO_2_RR on Au/C, CoPc/CNTs, and NiPc/CNTs. Partial current densities for CO production (**a**, **d**, **g**), corresponding KIE values (**b**, **e**, **h**), and Faradaic efficiencies (**c**, **f**, **i**) measured for the CO_2_RR on the Au/C (**a**–**c**), CoPc/CNTs (**d**–**f**), and NiPc/CNTs (**g**–**i**) electrodes in 1 M NaHCO_3_/H_2_O and 1 M NaDCO_3_/D_2_O electrolytes. The applied potentials were 100% *iR*-compensated using the current-interrupt method (NaHCO_3_/H_2_O: *R*_u_ = 6.9 ± 0.3 Ω, pH = 7.4; NaDCO_3_/D_2_O: *R*_u_ = 8.1 ± 0.7 Ω, pD = 7.8; geometric electrode area = 0.5 cm^2^). The error bars represent the standard deviation of three independent measurements. Source data are provided as a Source data file.

As shown in Fig. 3a, in both NaHCO_3_/H_2_O and NaDCO_3_/D_2_O electrolytes, the partial current density for CO production on the Au/C electrode gradually increased from 3.8 to 10.9 mA cm^−2^ as the potential shifted from −0.45 to −0.60 V versus RHE, resulting in KIE values of around 1 (Fig. 3b). Meanwhile, the CO Faradaic efficiency was 86–89% in the NaHCO_3_/H_2_O electrolyte, but increased to 95–97% in NaDCO_3_/D_2_O, as presented in Fig. 3c. This is because the D_2_ partial current densities in NaDCO_3_/D_2_O were significantly lower than H_2_ in NaHCO_3_/H_2_O, as shown in Supplementary Fig. 5, leading to a higher CO Faradaic efficiency in NaDCO_3_/D_2_O. This confirms the reliability of the KIE method for determining proton involvement in the rate-limiting step. The result indicates that the CO_2_RR on Au nanoparticles is limited by the CO_2_ adsorption step without proton involvement, in consistent with previous studies^38,40^. In contrast, the CoPc/CNTs electrode exhibited consistently higher CO partial current densities in NaHCO_3_/H_2_O than in NaDCO_3_/D_2_O over the potential range of −0.55 to −0.70 V versus RHE (Fig. 3d), leading to KIE values of 1.4–1.5 (Fig. 3e). The CO Faradaic efficiencies on CoPc/CNTs in NaDCO_3_/D_2_O were higher than those in NaHCO_3_/H_2_O (Fig. 3f), which is similar to the case of Au catalyst, due to the lower D_2_ production rates in NaDCO_3_/D_2_O. These results suggest that the protonation of adsorbed *CO_2_ is the rate-limiting step of CO_2_RR on CoPc/CNTs. Similarly, on the NiPc/CNTs electrode, CO production rate was generally higher in NaHCO_3_/H_2_O than in NaDCO_3_/D_2_O, yielding KIE values of 1.3–1.5, as shown in Fig. 3g, h. Meanwhile, NiPc/CNTs exhibited even higher selectivity for CO_2_RR, with negligible H_2_ detected (Fig. 3i). Therefore, the rate-limiting step on NiPc/CNTs is the protonation of adsorbed *CO_2_, consistent with previous studies showing a high energy barrier for this step at the Ni−N_4_ sites^20,38^. In addition, the KIE was evaluated for the concurrent HER during the CO_2_ electrolysis. As shown in Supplementary Fig. 5, the H_2_ partial current densities in NaHCO_3_/H_2_O were consistently higher than the corresponding D_2_ partial current densities in NaDCO_3_/D_2_O for all catalysts, demonstrating the reliability of the KIE method in probing proton involvement in the rate-limiting step.

The above studies were performed in near-neutral electrolytes, while electrolyte pH may also influence the rate-limiting step of CO_2_RR on metal phthalocyanines. Therefore, we further examined the rate-limiting step of CO_2_RR on CoPc/CNTs in acidic media (1 M NaClO_4_/H_2_O and 1 M NaClO_4_/D_2_O, pH/pD = 2) using KIE analysis. As exhibited in Supplementary Fig. 6, the partial current densities for CO production in NaClO_4_/H_2_O were consistently higher than those in NaClO_4_/D_2_O, yielding KIE values of 1.3–1.7. This indicates that the rate-limiting step of CO_2_RR on metal phthalocyanines is not altered by pH variation. In addition, methanol can be formed as a liquid product during CO_2_RR on CoPc/CNTs^31^, which was detected in 1 M NaHCO_3_ electrolyte at more negative potentials (<−0.80 V versus RHE), as shown in Supplementary Fig. 7. However, methanol formation occurs only at high overpotentials with relatively low selectivity compared to CO production. The possible divergence between our methanol production performance and prior reports likely arises from the high sensitivity of this pathway to experimental parameters, such as CoPc-to-CNT mass ratio, catalyst loading, and CO_2_ partial pressure^31,33–35^. Given that the present work is centered on CO_2_RR to CO on metal phthalocyanines and aims to elucidate the fundamental rate-limiting steps governing this primary reaction pathway, the following studies will focus on the CO_2_-to-CO conversion.

### Effect of molecular dispersion state on the rate-limiting step

**Fig. 4:**
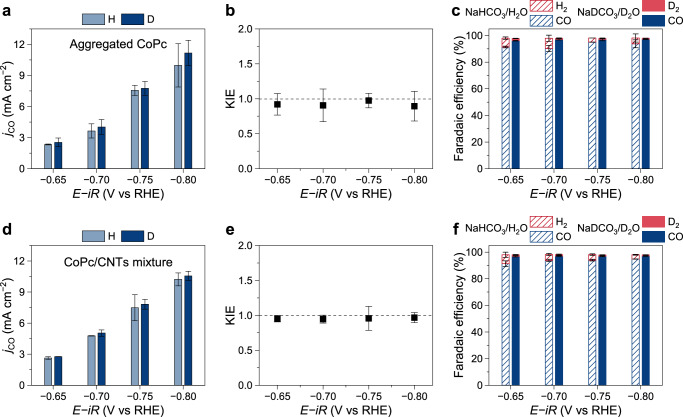
Effect of molecular dispersion on the rate-limiting step of CO_2_RR. Partial current densities for CO production (**a**, **d**), corresponding KIE values (**b**, **e**), and Faradaic efficiencies (**c**, **f**) for CO_2_RR on the aggregated CoPc electrode (**a**–**c**) and the CoPc/CNTs mixture electrode (**d**–**f**), measured in 1 M NaHCO_3_/H_2_O and 1 M NaDCO_3_/D_2_O electrolytes. The applied potentials were 100% *iR*-compensated using the current-interrupt method (NaHCO_3_/H_2_O: *R*_u_ = 6.9 ± 0.3 Ω, pH = 7.4; NaDCO_3_/D_2_O: *R*_u_ = 8.1 ± 0.7 Ω, pD = 7.8; geometric electrode area = 0.5 cm^2^). The error bars represent the standard deviation of three independent measurements. Source data are provided as a Source data file.

As previously discussed, the rate-limiting step of CO_2_RR on CoPc catalyst remains controversial, with prior studies also identifying CO_2_ adsorption as the rate-limiting step using the KIE method^37^. This discrepancy could stem from variations in CoPc immobilization methods, which lead to different dispersion states that have been reported to influence CO_2_RR activity and selectivity^18,33^. For instance, the commonly used method of directly depositing CoPc dissolved in *N*,*N*-Dimethylformamide (DMF) onto substrates may cause aggregation of CoPc molecules due to the low surface area of substrates. To test this hypothesis, we prepared an additional CoPc electrode by directly depositing a DMF solution of CoPc onto an AvCarb GDS2230 carbon substrate. SEM characterization was then performed to examine its morphology. As shown in Supplementary Fig. 8, the electrode exhibited newly formed nanoparticles and nanoflakes, ranging from submicrometers to a few micrometers, on the substrate, suggesting the formation of CoPc aggregates. The aggregated CoPc electrode was then evaluated for CO_2_RR, which showed a lower activity compared to the above-tested CoPc/CNTs electrode, as exhibited in Fig. 4a. More importantly, the CO partial current densities in NaHCO_3_/H_2_O and NaDCO_3_/D_2_O were nearly identical, resulting in KIE values of around 1, as shown in Fig. 4b. The CO Faradaic efficiencies were all greater than 90% (Fig. 4c), suggesting that CO_2_RR was the dominant reaction. In contrast, the concurrent HER activities in the two electrolytes still showed a notable difference, yielding KIE values greater than 3 and indicating reliable KIE measurements (Supplementary Fig. 9). The results confirm that the rate-limiting step of CO_2_RR on the aggregated CoPc electrode does not involve protons and should be the CO_2_ adsorption step.

To further examine the possible influence of CNTs on the above-observed difference in the rate-limiting step, we prepared a control sample by mixing CoPc powders and CNTs, which will hereafter be referred to as the “CoPc/CNTs mixture” sample. As shown in Supplementary Fig. 10, SEM imaging revealed the formation of CoPc aggregates of a few micrometers in size, with their crystalline structure confirmed by the XRD pattern. The CoPc/CNTs mixture sample was then deposited onto an AvCarb GDS2230 substrate to form an electrode and evaluated for the KIE in CO_2_RR. As exhibited in Fig. 4d–f, the sample showed comparable CO_2_RR performance, including the partial current density and Faradaic efficiency for CO production, and the similar KIE values (~1) indicate that the CO_2_RR has the same rate-limiting step—CO_2_ adsorption. Meanwhile, the concurrent HER showed a typical KIE on the CoPc/CNTs mixture electrode (Supplementary Fig. 11). Collectively, the differing rate-limiting steps of CO_2_RR on CoPc molecules can be attributed to their distinct dispersion states, which may account for discrepancies among previous studies. This correlation likely arises from variations in the electric field around the Co centers in CoPc molecules, as will be further discussed below.

Prior to investigating the underlying mechanism, it is essential to establish that the observed differences in CO_2_RR activity between NaHCO_3_/H_2_O and NaDCO_3_/D_2_O do not arise from extrinsic factors, such as differences in CO_2_ solubility or the p*K*_a_ values of H_2_O versus D_2_O. First, the molar solubilities of CO_2_ in H_2_O and D_2_O at 25 °C and 1 atm are both ~33.7 mmol L^−^^1^^41^, which cannot account for the pronounced CO_2_RR activity differences. Moreover, in 1 M NaHCO_3_/H_2_O and 1 M NaDCO_3_/D_2_O electrolytes, the equilibrium between dissolved CO_2_ and bicarbonate species (CO_2_ + H_2_O ⇌ H⁺ + HCO_3_^−^, CO_2_ + D_2_O ⇌ D^+^ + DCO_3_^−^) can continuously buffer and replenish CO_2_ near the cathode, ensuring a sustained and comparable CO_2_ supply for the CO_2_RR. Consequently, CO_2_ solubility or availability can be excluded as the origin of the observed activity differences in the KIE studies.

Second, H_2_O and D_2_O exhibit a modest difference in their acid–base properties (p*K*_w_ = 14.00 for H_2_O and 14.86 for D_2_O), resulting in bulk pH/pD values of 7.4 and 7.8 for CO_2_-saturated 1 M NaHCO_3_ and NaDCO_3_ electrolytes, respectively. In the above measurements, all potentials were converted to the RHE scale using the corresponding bulk pH, thereby accounting for pH-dependent thermodynamic effects in the comparison of CO_2_RR activities. Nevertheless, local pH may change during reactions and influence reaction kinetics. To isolate genuine isotope effects from possible pH-induced artifacts, we employed in situ scanning electrochemical microscopy (SECM) to probe local pH under *operando* conditions. As shown in Supplementary Fig. 12, a customized SECM setup^42^, equipped with an Au nanoelectrode tip whose exposed apex was functionalized with 4-hydroxylaminothiophenol (4-HATP)/4-nitrosothiophenol (4-NSTP), was used to measure the local pH near the cathode^43,44^. A correlation between the mid-peak potential of the 4-HATP/4-NSTP anodic peak and the electrolyte pH was established as a calibration curve (Supplementary Fig. 12). Using this approach, we quantified the local pH near the CoPc/CNTs electrode under various CO_2_RR current densities, as presented in Supplementary Fig. 13. While a slight increase in local pH was observed during CO_2_RR in both electrolytes, the relative difference between them remained ~0.4 pH units, consistent with the initial bulk pH difference (7.4 versus 7.8). Because this initial difference was already accounted for in the RHE-referenced potentials, the minor local pH shifts do not exert any additional influence on the KIE analysis.

### Mechanisms underlying distinct rate-limiting steps

The above electrochemical measurements and KIE analysis indicate that the rate-limiting step for CO_2_RR to CO on CoPc molecules depends on their dispersion state. For molecularly dispersed CoPc on CNTs, the reaction is limited by the protonation of *CO_2_, whereas for aggregated CoPc, CO_2_ adsorption becomes the rate-limiting step. We hypothesize that the shift of the rate-limiting step on aggregated CoPc arises from a weakened interfacial electric field at the Co active sites. Bulk CoPc, as an organic semiconductor, exhibits low conductivity and pronounced dielectric behavior^45,46^, which limits penetration of the applied electric field into thick CoPc aggregates^33^. Consequently, the Co active sites in aggregated CoPc experience a weaker electric field than those in molecularly dispersed CoPc, reducing the driving force for initial CO_2_ activation and shifting the rate-limiting step. The change in the electric field at Co sites upon CoPc aggregation was revealed by Stark tuning, based on the potential-dependent frequency shifts of reaction intermediates in sum frequency generation spectra^33^. Here, we perform further mechanistic investigations to validate this hypothesis.

**Fig. 5:**
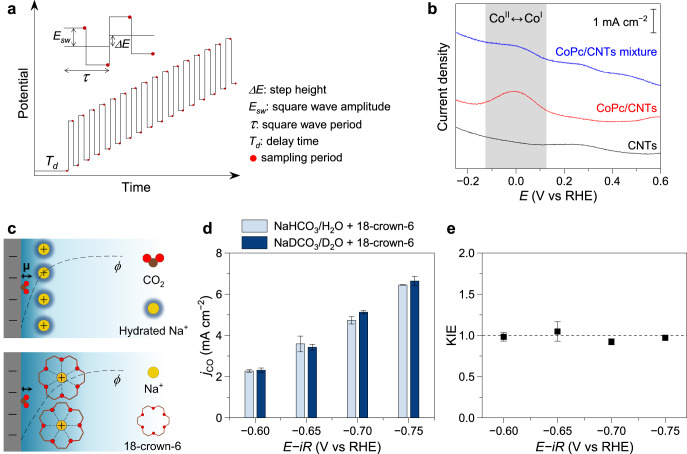
Mechanistic insights into distinct rate-limiting steps. **a** Schematic illustration showing the potential modulation in SWV. **b** SWV curves recorded on electrodes with different dispersion states of CoPc molecules. Step height: 2 mV; amplitude: 15 mV; frequency: 25 Hz. The applied potentials in (**b**) were not *iR*-compensated. **c** Schematic illustration showing the effect of 18-crown-6 on the interfacial electric field. **d** Partial current densities for CO production during CO_2_RR on CoPc/CNTs in NaHCO_3_/H_2_O + 18-crown-6 and in NaDCO_3_/D_2_O + 18-crown-6 electrolytes. **e** Corresponding KIE values. The applied potentials in (**d**, **e**) were 100% *iR*-compensated using the current-interrupt method (NaHCO_3_/H_2_O + 18-crown-6: *R*_u_ = 8.7 ± 0.3 Ω, pH = 7.4; NaDCO_3_/D_2_O + 18-crown-6: *R*_u_ = 9.2 ± 0.3 Ω, pD = 7.8; geometric electrode area = 0.5 cm^2^). The error bars represent the standard deviation of three independent measurements. Source data are provided as a Source data file.

First, we compared the Co(II)/Co(I) redox responses of the CoPc/CNTs electrode and the CoPc/CNTs mixture electrode to examine the potential-screening effect due to CoPc aggregation. Because CNTs can introduce substantial capacitive currents and parasitic Faradaic contributions, we employed square-wave voltammetry (SWV), which can suppress the charging currents while providing high sensitivity^47,48^. A representative SWV waveform is illustrated in Fig. 5a. As shown in Fig. 5b, molecularly dispersed CoPc on CNTs experiences a strong local electric field, resulting in a pronounced and well-defined Co(II)/Co(I) redox peak at around 0 V vs RHE, consistent with previous reports^34^. In contrast, despite identical CoPc loadings, the redox peak of the aggregated CoPc is significantly attenuated compared to that of the molecularly dispersed CoPc, indicating a suppression of the Co(II)/Co(I) redox response due to aggregation. When a potential is applied to the electrode, the electric field does not fully penetrate the aggregates, limiting effective potential transmission to the Co active sites and resulting in a substantially attenuated redox signal.

To further examine whether attenuation of the electric field shifts the rate-limiting step to CO_2_ adsorption, we intentionally reduced the local electric field around molecularly dispersed CoPc by chelating Na⁺ in the electrolyte using the crown ether 18-crown-6^49^. Chelation of Na⁺ effectively increases the apparent cation size and expands the thickness of the outer Helmholtz layer^50,51^, thereby reducing the potential gradient and interfacial electric field, as schematically illustrated in Fig. 5c. KIE studies were then performed on the CoPc/CNTs electrode in 1 M NaHCO_3_/H_2_O + 18-crown-6 and in 1 M NaDCO_3_/D_2_O + 18-crown-6 electrolytes. As shown in Fig. 5d, the partial current densities for CO production greatly decreased in the NaHCO_3_ + 18-crown-6 electrolyte compared to those in NaHCO_3_ (Fig. 3d), indicating reduced activity due to the weakened electric field. Importantly, the CO_2_RR activities in crown-ether-containing H_2_O and D_2_O electrolytes were nearly identical, giving KIE values of ~1 (Fig. 5e). These results indicate that upon weakening the electric field, the rate-limiting step shifts to CO_2_ adsorption even for molecularly dispersed CoPc. In contrast, KIE values of ~3 were observed for the concurrent HER (Supplementary Fig. 14), confirming the reliability of KIE analysis in the presence of crown ether. Overall, these findings demonstrate that the Co active sites in aggregated CoPc experience a weakened electric field so that CO_2_ adsorption becomes rate-limiting. This provides a clear mechanistic explanation for the dependence of the CO_2_RR rate-limiting step on the dispersion state of CoPc.

### Effect of electrolyte anions on the CO_2_RR with CoPc/CNTs

**Fig. 6:**
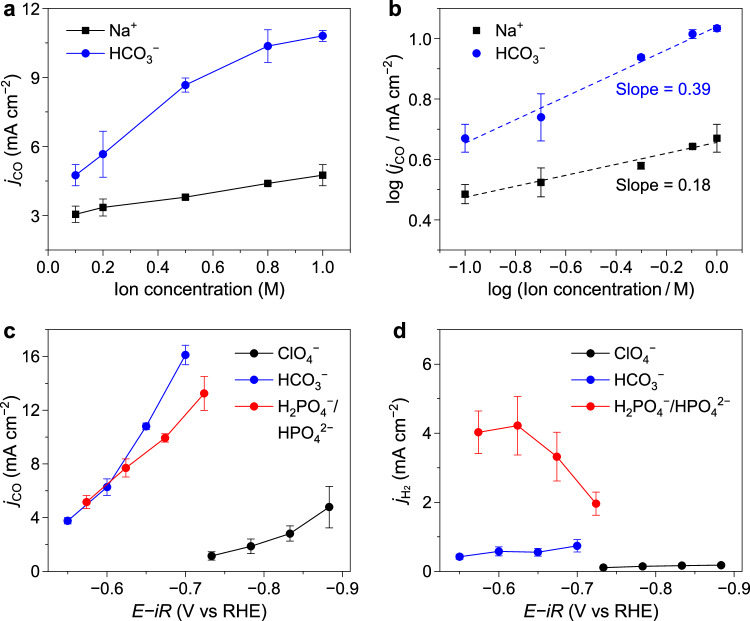
Effect of electrolyte ions on CO_2_RR with CoPc/CNTs. Partial current densities for CO production during CO_2_RR at −0.65 V versus RHE in electrolytes with varying concentrations of Na^+^ and HCO_3_^−^ (**a**), and corresponding logarithmic plots (**b**) to determine reaction orders. Partial current densities for CO (**c**) and H_2_ (**d**) production during CO_2_RR in electrolytes containing 1 M Na^+^ paired with different anions. The applied potentials in (**c**, **d**) were 100% *iR*-compensated using the current-interrupt method (ClO_4_^−^: *R*_u_ = 7.2 ± 0.1 Ω, pH = 4.3; HCO_3_^−^: *R*_u_ = 6.9 ± 0.3 Ω, pH = 7.4; H_2_PO_4_^−^/HPO_4_^2−^: *R*_u_ = 11.6 ± 0.4 Ω, pH = 6.6; geometric electrode area = 0.5 cm^2^). The error bars represent the standard deviation of three independent measurements. Source data are provided as a Source data file.

The above insights into the rate-limiting step of CO_2_RR on CoPc catalysts motivated an investigation of electrolyte anion effects, which have been relatively overlooked compared with cations. It is well-established that on typical metal catalysts, such as Au and Ag, CO_2_RR relies on metal cations to facilitate the rate-limiting CO_2_ adsorption by stabilizing adsorbed *CO_2_ via electrostatic interaction, resulting in an apparent dependence on the cation identity and concentration^52–54^. In contrast, anions, including the widely used HCO_3_^−^, act more as a spectator without involvement in the rate-limiting step, as evidenced by the zero reaction order measured for CO_2_RR on Au^55,56^. Given the distinct rate-limiting step on CoPc/CNTs, the effects of cations and anions on CO_2_RR are expected to differ from those observed on metal catalysts. We thus evaluated the dependence of CO_2_RR activity on the concentrations of Na^+^ and HCO_3_^−^, respectively. To ensure a rigorous comparison, the concentration of HCO_3_^−^ was held constant while varying Na^+^, and vice versa. Electrolyte electroneutrality was maintained by adding ClO_4_^−^ as necessary. As shown in Fig. 6a, the CO partial current density on CoPc/CNTs at −0.65 V versus RHE increased from 3.1 to 4.7 mA cm^−2^ as the Na^+^ concentration increased from 0.1 to 1.0 M, yielding a reaction order of 0.18 (Fig. 6b). This cation effect likely arises from the promoted hydrolysis of the Na^+^ hydration shell^32^, which can facilitate the rate-limiting protonation step of adsorbed *CO_2_. Interestingly, the CO_2_RR activity on CoPc/CNTs also increased with the HCO_3_^−^ concentration, even more pronounced than that for Na^+^, resulting in a higher reaction order of 0.39. These results indicate that the CO_2_RR on CoPc/CNTs exhibits a stronger dependence on HCO_3_^−^ anion than on Na^+^ cation, in sharp contrast to the behavior observed for metal catalysts^55,56^. This should be attributed to the promotional effect of HCO_3_^−^ on the rate-limiting protonation step of adsorbed *CO_2_ on CoPc/CNTs, as HCO_3_^−^ can act as a proton donor in both CO_2_RR and HER, as reported in previous studies^57–59^.

To further examine this effect, we selected anions with different proton-donating abilities, including ClO_4_^−^ (non-proton-donating), HCO_3_^−^ (p*K*_a_ ~ 10.3), and H_2_PO_4_^−^/HPO_4_^2−^ (p*K*_a_ ~ 7.2), thus to compare their influence on the CO_2_RR with CoPc/CNTs. The comparative study was performed in electrolytes containing 1 M Na^+^ paired with different anions. As shown in Fig. 6c, the CO_2_RR activity within the potential range of −0.55 to −0.90 versus RHE follows the trend: ClO_4_^−^ *<* H_2_PO_4_^−^/HPO_4_^2−^ ~ HCO_3_^−^, reflecting differences in proton-donating ability, though not strictly mirroring their p*K*_a_ values. This can be explained by including the concurrent HER activity during the electrolysis, which follows the trend: ClO_4_^−^ *<* HCO_3_^−^ *<* H_2_PO_4_^−^/HPO_4_^2−^ (Fig. 6d). As a result, the H_2_PO_4_^−^/HPO_4_^2−^ electrolyte showed the highest total current density (Supplementary Fig. 15), consistent with its stronger proton-donating capacity, although a larger fraction of the available protons was consumed by the HER, leading to lower Faradaic efficiencies for CO production. In addition, the CO_2_RR activity with HCO_3_^−^ became higher than that with H_2_PO_4_^−^/HPO_4_^2−^ at more negative potentials (Fig. 6c). This is attributed to the distinct role of HCO_3_^−^ as a CO_2_ shuttle, which can increase the local concentration of CO_2_ and the CO_2_RR activity^36,60^, especially when the mass transport limit is approached at high reaction rates in H-cell. These results indicate that the CO_2_RR activity on CoPc/CNTs increases with the proton donation ability of electrolyte anions, confirming their role as a proton donor in the rate-limiting protonation step of adsorbed *CO_2_.

The proton-donating ability of the anions may not only facilitate protonation steps in CO_2_RR, but also provide buffering capacity to mitigate local pH changes during reactions^61,62^. To assess the contribution of such mechanistic factors, we employed the SECM with pH-sensitive probes to monitor the local pH during CO_2_RR in different electrolytes. As shown in Supplementary Fig. 16a, the local pH near the CoPc/CNTs electrode during CO_2_RR in NaClO_4_ increased markedly, from an initial bulk pH of 4.3 to a local pH of 8.6 at a current density of 5.0 mA cm^−2^, reflecting the absence of buffering capacity in NaClO_4_. In contrast, the pH rise in NaHCO_3_ was much smaller (Supplementary Fig. 13) due to its buffering nature. To account for pH-dependent thermodynamic effects when comparing CO_2_RR activities, we recalculated the activities using RHE-referenced potentials corrected by the measured local pH values (Supplementary Fig. 16b). Even after this correction, the CO_2_RR activities in NaHCO_3_ remained consistently higher than those in NaClO_4_, indicating that differences in buffering or local pH do not explain the variation in CO_2_RR activity among the anions. Instead, the higher CO_2_RR activity in NaHCO_3_ is mainly attributed to enhanced *CO_2_ protonation on CoPc/CNTs due to the proton-donating ability of the HCO_3_^−^ anion.

These findings suggest a strategy for enhancing CO_2_ electrolysis systems with a rate-limiting protonation step through the use of proton-donating anions^61,62^. To further demonstrate this anion strategy and its applicability, we evaluated CO_2_ electrolysis in presence of different anions using a flow cell equipped with gas-diffusion electrodes (GDEs), where the CO_2_RR current density can be drastically increased due to enhanced mass transport^14^. Indeed, as shown in Supplementary Fig. 17, the NaHCO_3_ electrolyte achieved the highest activity for CO_2_RR on CoPc/CNTs, followed by NaH_2_PO_4_/Na_2_HPO_4_ and NaClO_4_. Moreover, given the negatively charged cathode during CO_2_RR, we expect electrolyte cations capable of donating protons to be more effective in promoting the rate-limiting protonation step. Such cations can enter and accumulate in the electric double layer, allowing direct interactions with surface-bound intermediates and thereby enabling more efficient proton delivery to facilitate *CO_2_ protonation.

## Discussion

The above studies focus on enhancing CO_2_RR on CoPc with a rate-limiting protonation step by leveraging the proton-donating ability of electrolyte ions. More broadly, the methodology demonstrated here, which identifies whether the CO_2_RR rate-limiting step involves proton transfer through KIE analysis and then optimizes the electrolyte accordingly, is applicable to other metal phthalocyanines with different metal centers (Ni, Fe, Cu, Mn) and to related molecular catalysts such as porphyrins and quaterpyridines. For catalysts with a protonation-limited pathway, CO_2_RR performance can be enhanced by selecting electrolyte ions with appropriate proton-donating ability. Conversely, for systems where CO_2_ adsorption is rate-limiting, CO_2_RR activity can be improved by strengthening the interfacial electric field, for example, through the use of partially or weakly solvated cations^54^. Together, these insights provide generalizable guidance for the rational design of efficient CO_2_ electrolysis systems.

In summary, we investigated the rate-limiting step of CO_2_RR to CO on metal phthalocyanines with Au as a reference. Using electrochemical measurements and KIE analysis, we identified the protonation of adsorbed *CO_2_ as the rate-limiting step on molecularly dispersed CoPc and NiPc supported on CNTs. We further revealed that the rate-limiting step depends on the dispersion state of metal phthalocyanine molecules: on aggregated CoPc, it shifts to the CO_2_ adsorption due to a weakened interfacial electric field at the Co active sites. These mechanistic insights also highlight a previously overlooked effect of electrolyte anions: HCO_3_^−^ strongly promotes the CO_2_RR on CoPc/CNTs by acting as a proton donor in the rate-limiting protonation step, as confirmed by a comparative study using anions with different proton donation abilities. Our study elucidates the rate-limiting step of CO_2_RR on metal phthalocyanine catalysts, establishes molecular dispersion as a key structural parameter, and provides guidance for the rational design of efficient molecular electrocatalysts.

## Methods

### Chemicals

Ethanol (99.5%, anhydrous, 200 proof), 2-propanol (HPLC), *N*,*N*-Dimethylformamide (DMF, Certified ACS), hydrochloric acid (HCl, 34–37%, trace-metal grade), sulfuric acid (H_2_SO_4_, 98%), cobalt (Ⅱ) phthalocyanine (CoPc, C_32_H_16_N_8_Co), tetrachloroauric acid trihydrate (HAuCl_4_·3H_2_O, Certified ACS), sodium citrate (C_6_H_5_Na_3_O_7_, 99+%), tannic acid (C_76_H_52_O_46_, Certified ACS), hydrogen peroxide (H_2_O_2_, 30%, Certified ACS), sodium carbonate (Na_2_CO_3_, 99.95%), sodium bicarbonate (NaHCO_3_, 99.7+%), sodium perchlorate (NaClO_4_, ACS Grade), sodium phosphate monobasic monohydrate (NaH_2_PO_4_·H_2_O, 99+%), sodium phosphate dibasic heptahydrate (Na_2_HPO_4_·7H_2_O, 99+%), deuterium oxide (D_2_O, 99.8 atom% D), titanium gauze (80 mesh), iridium(IV) chloride (IrCl_4_·xH_2_O), 18-Crown-6 (99%), and Au wires (0.5 mm diameter, 99.95%) were purchased from Fisher Scientific. Nickel (Ⅱ) phthalocyanine (NiPc, C_32_H_16_N_8_Ni), 4-nitrothiophenol (4-NTP), and Nafion perfluorinated resin solution (5 wt%) were purchased from Sigma Aldrich. Multi-walled carbon nanotubes (US4403, purity: >99.9 wt%, outside diameter: 10–20 nm) were purchased from US Research Nanomaterials, Inc. Nafion 115 and 117 membranes, Vulcan XC-72 carbon black, and AvCarb GDS2230 carbon substrates were purchased from Fuel Cell Store. Ag/AgCl reference electrodes (MF-2056, 3 M KCl) were purchased from BASi Research Products. Leak-free Ag/AgCl reference electrodes (LF-1-100) were purchased from Innovative Instruments. Ar (99.999%), H_2_ (99.999%), CO_2_ (99.995%), and CO (99.9%) gases were purchased from Airgas. Deionized water with a specific resistance of 18.2 MΩ cm was used in all experiments.

### Preparation of catalysts and electrodes

The CoPc/CNTs sample was prepared using a method reported in previous studies^22,31^. Commercial CNTs were purified by heat treatment in air for 5 h, followed by sonication in a 5 wt% HCl solution for 30 min. The purified CNTs were collected by filtration, washed with deionized water, and dried at 60 °C. 50 mg of the purified CNTs and 1.5 mg of CoPc were each dispersed in 20 mL DMF and sonicated for 1 h. The two dispersions were then mixed, sonicated for 30 min, and stirred at room temperature (25 °C) for 20 h. The resulting solid was collected by centrifugation, washed with DMF and ethanol, and lyophilized to obtain the CoPc/CNTs sample. The CoPc content in the sample was determined to be 0.96 wt% using inductively coupled plasma optical emission spectroscopy (ICP-OES). The NiPc/CNTs sample was prepared using the same procedure as for CoPc/CNTs, with NiPc substituted for CoPc. The NiPc content in the NiPc/CNTs sample was determined to be 0.84 wt% using ICP-OES. To prepare the CoPc/CNTs mixture sample, 49.5 mg of purified CNTs and 0.5 mg CoPc were ground together in a motor for 30 min. Catalyst inks were prepared by mixing 5.6 mg of each sample (CoPc/CNTs, NiPc/CNTs, and the CoPc/CNTs mixture) with 1.6 mL of 2-propanol and 45 µL of Nafion solution (5 wt%), followed by sonication for 1 h. The inks were then drop-cast onto an AvCarb GDS2230 carbon substrate (0.71 × 0.71 cm^2^; same dimensions for all electrodes used in the H-cell studies) until a loading of 0.5 mg cm^−2^ (geometric area basis) was reached, resulting in the final electrodes. The aggregated CoPc electrode was prepared by drop-casting a DMF solution of CoPc with a concentration of 0.1 mg mL^−1^ onto an AvCarb GDS2230 carbon substrate until a loading of 0.005 mg cm^−2^ was reached. The CoPc loading on the CoPc/CNTs, CoPc/CNTs mixture, and aggregated CoPc electrodes was kept nearly identical for a rigorous comparison of their catalytic performances.

The Au/C nanopowder was prepared using a two-step method, including the synthesis of Au nanoparticles and subsequent loading onto carbon support. The Au nanoparticles were synthesized following the procedure described in a previous study^63^. 1 mL of tetrachloroauric acid (HAuCl_4_, 25 mM) was injected into a mixed solution containing 150 mL of sodium citrate (2.2 mM), 0.1 mL of tannic acid (2.5 mM), and 1 mL of Na_2_CO_3_ (150 mM) at 50 °C. The resulting solid was collected by filtration, washed with deionized water, and freeze-dried to obtain the Au nanoparticles. 45 mg of Vulcan X-72 carbon black and 5 mg of the prepared Au nanoparticles were dispersed in 20 mL of deionized water and sonicated for 1 h. The dispersion was filtered, and the precipitate was washed with deionized water and dried at 60 °C overnight to obtain the Au/C sample. To prepare Au/C electrodes, 5.6 mg of the Au/C sample, 1.6 mL of 2-propanol, and 45 µL of Nafion solution (5 wt%) were mixed and sonicated for 1 h to form a catalyst ink. The ink was then drop-cast onto an AvCarb GDS2230 carbon substrate until a loading of 1.0 mg cm^−2^ was reached.

### Materials characterization

SEM images were acquired using a ZEISS Ultra-55 FEG scanning electron microscope. TEM images, STEM images, and corresponding EDS mapping were acquired using a FEI Tecnai F30 transmission electron microscope with a field emission gun operated at 200 kV. XRD patterns were collected using a PANalytical Empyrean diffractometer with a 1.8 KW copper X-ray tube. XPS spectra were acquired using a Thermo Scientific ESCALAB XI^+^ X-ray photoelectron spectrometer with an Al Kα X-ray source (1486.67 eV).

### Electrochemical measurements

All electrochemical measurements were performed using a Gamry Interface 1000 potentiostat with a gas-tight two-compartment electrochemical cell (H-cell, Supplementary Fig. 4) at room temperature (25 °C). The above prepared electrodes were used as cathodes, and an Ag/AgCl electrode and a piece of Pt gauze were used as the reference and counter electrodes, respectively. A Nafion 115 membrane (thickness: 127 µm) was used to separate the cathode and anode chambers. Prior to use, Nafion 115 membranes were sequentially heat-treated at 80 °C in 5% H_2_O_2_, 0.5 M H_2_SO_4_, and deionized water for 1 h each, then thoroughly rinsed and stored immersed in deionized water until use. The catholyte and anolyte each contained 15 mL of electrolyte. Four electrolyte compositions were used: 1 M NaHCO_3_, 1 M NaDCO_3_ in D_2_O (KIE experiments), 1 M NaClO_4_, and a phosphate buffer (0.3 M NaH_2_PO_4_ + 0.35 M Na_2_HPO_4_). Electrolytes were prepared in H_2_O unless otherwise noted. The NaDCO_3_/D_2_O electrolyte was prepared by bubbling CO_2_ gas through a Na_2_CO_3_ solution in D_2_O. Prior to CO_2_RR testing, the catholyte was purged with CO_2_ for 30 min and then continuously purged at a rate of 5 sccm during bulk electrolysis while stirring at 600 rpm; the CO_2_ gas flow rate was controlled and monitored using an Alicat Scientific mass flow controller. The GDE-cell studies were carried out using a home-built GDE flow cell^14^, which consists of a Ti current collector with interdigitated gas-diffusion channels, a cathodic GDE with the catalyst layer deposited on AvCarb GDS2230 substrate, a 3D-printed chamber with ports for electrolyte flow and reference electrode, and an IrO_x_/Ti gauze anode inserted in a pocket of another Ti current collector. A leak-free Ag/AgCl electrode was used as the reference electrode. The catholyte and anolyte (15 mL each) were circulated using peristaltic pumps at 2.0 mL min^−1^. All potentials were 100% *iR*-compensated using the current-interrupt method (unless otherwise specified) and are reported versus RHE. Potentials were measured versus an Ag/AgCl (3 M KCl) reference electrode and converted to the RHE scale using *E*_RHE_ = *E*_Ag/AgCl_ + 0.210 V + 0.0591 × pH. The reference electrode was calibrated using a standard hydrogen electrode before measurements. The reported current densities were normalized to the geometric surface areas of the electrodes. During CO_2_ electrolysis, gas-phase products were quantified by a gas chromatograph (SRI Multiple Gas Analyzer #5) equipped with molecular sieve 5A and HayeSep D columns with Ar carrier gas. Liquid-phase products in the catholyte were analyzed by ^1^H NMR spectroscopy (Bruker Avance III 500 MHz) after electrolysis.

### Calculation of CO_2_RR activity and Faradaic efficiencies

The partial current densities for CO and H_2_ production were calculated from the GC peak area as follows:

1 $${j}_{{{{\rm{partial}}}}}=\frac{{{{\rm{peak}}}}\,{{{\rm{area}}}}}{\alpha }\times ({{{\rm{gas}}}}\,{{{\rm{flow}}}}\,{{{\rm{rate}}}})\times \frac{nFp}{RT}\times {({{{\rm{electrode}}}}\,{{{\rm{area}}}})}^{-1}$$

where *α* is a gas-dependent conversion factor determined by GC calibration, *n* is the number of electrons transferred for the product, *F* is the Faraday constant, *R* is the ideal gas constant, *p* and *T* are the gas pressure and temperature, and the gas flow rate is the CO_2_ feed rate delivered to the electrochemical cell (H-cell or GDE flow cell). The Faradaic efficiencies for CO and H_2_ production were calculated by dividing the corresponding *j*_partial_ by the total current density.

### Probing local pH using SECM

Local pH measurements were performed using a CH Instruments 920D SECM instrument equipped with three-directional stepper motor and piezoelectric actuators for coarse control and fine positioning of the SECM probe, respectively. A bipotentiostat was used to control the potentials applied to the substrate working electrode (CoPc/CNTs on glassy carbon) and the SECM probe. The Au nanoelectrode SECM probe was fabricated by electrochemical etching of an Au wire to form a nanoscale apex radius, followed by electropolymerization coating to insulate the shaft while leaving the Au apex exposed^42^. The exposed Au apex was functionalized with a self-assembled monolayer of 4-nitrothiophenol (4-NTP), which was then electrochemically converted into the 4-HATP/4-NSTP redox couple^43,44^. The Au nanoelectrode design ensures that only the tip apex serves as the active sensing area, enabling local pH measurements with minimal perturbation to the reaction environment. To maintain consistent probe–substrate separation across measurements, the Au nanoelectrode probe was first brought to electrical contact with the substrate using the piezoelectric fine-approach routine and then retracted by 1 μm and held at that position during pH measurements under CO_2_RR conditions. Local pH was quantified by recording cyclic voltammograms at the Au nanoelectrode probe and monitoring the Nernstian shift of the mid-peak potential of the surface-bound 4-HATP/4-NSTP redox couple (Supplementary Fig. 12).

## Supplementary information

Supplementary Information

Transparent Peer Review file

## Source data

Source data

## Data Availability

The data that support the findings of this study are available in the article and its Supplementary Information file. Source data are provided with this paper.
