# Supplementary material for: Elucidating the rate-limiting step of CO2 electroreduction on metal phthalocyanines
Source: Nat Commun. 2026 Mar 10;17:3720. doi: 10.1038/s41467-026-70445-9 (PMC13103363; doi:10.1038/s41467-026-70445-9)
Supplement: Supplementary file 1 — Supplementary Information [file 41467_2026_70445_MOESM1_ESM.pdf]

Supplementary Information

Elucidating the rate-limiting step of CO_2 electroreduction on metal phthalocyanines

Zhuanghe Ren[1], Kaige Shi[1], Zhen Meng[2], Thomas Egan[2], Talat S. Rahman[1,3] & Xiaofeng Feng[1,2,3,4]*

[1]Department of Physics, University of Central Florida, Orlando, FL 32816, USA.

[2]Department of Chemistry, University of Central Florida, Orlando, FL 32816, USA.

[3]Renewable Energy and Chemical Transformations (REACT) Cluster, University of Central Florida, Orlando, FL 32816, USA.

[4]Department of Materials Science and Engineering, University of Central Florida, Orlando, FL 32816, USA.

*Corresponding author. E-mail: Xiaofeng.Feng@ucf.edu

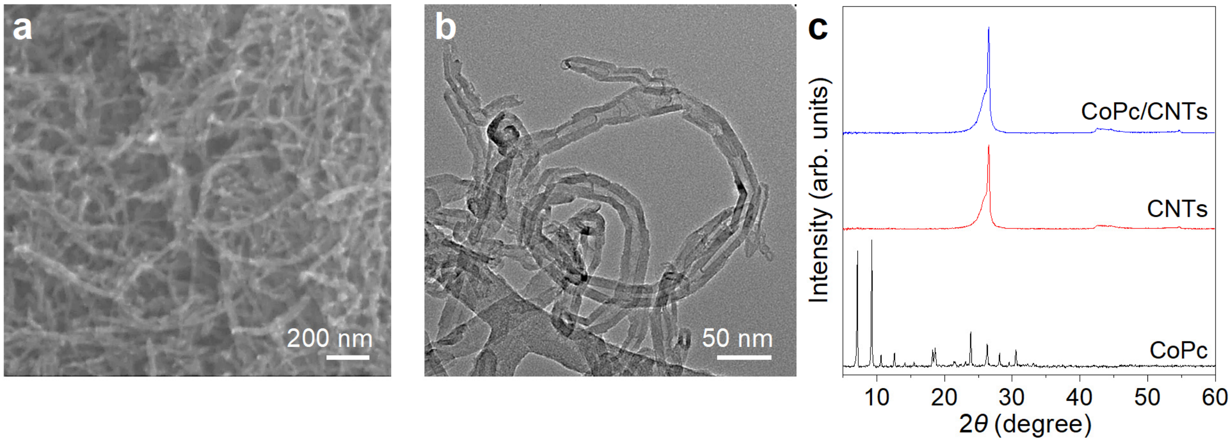

Supplementary Fig. 1. Characterization of the CoPc/CNTs sample. **a,** SEM image and **b,** TEM image of the CoPc/CNTs sample. **c,** XRD pattern of the CoPc/CNTs sample, with CNTs and CoPc as references. Source data are provided as a Source Data file.

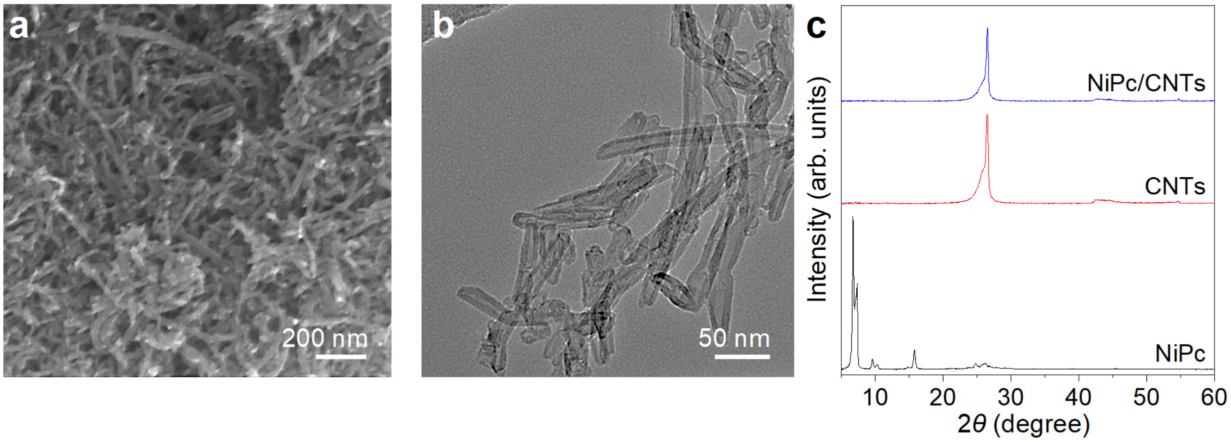

Supplementary Fig. 2. Characterization of the NiPc/CNTs sample. **a,** SEM image and **b,** TEM image of the NiPc/CNTs sample. **c,** XRD pattern of the NiPc/CNTs sample, with CNTs and NiPc as references. Source data are provided as a Source Data file.

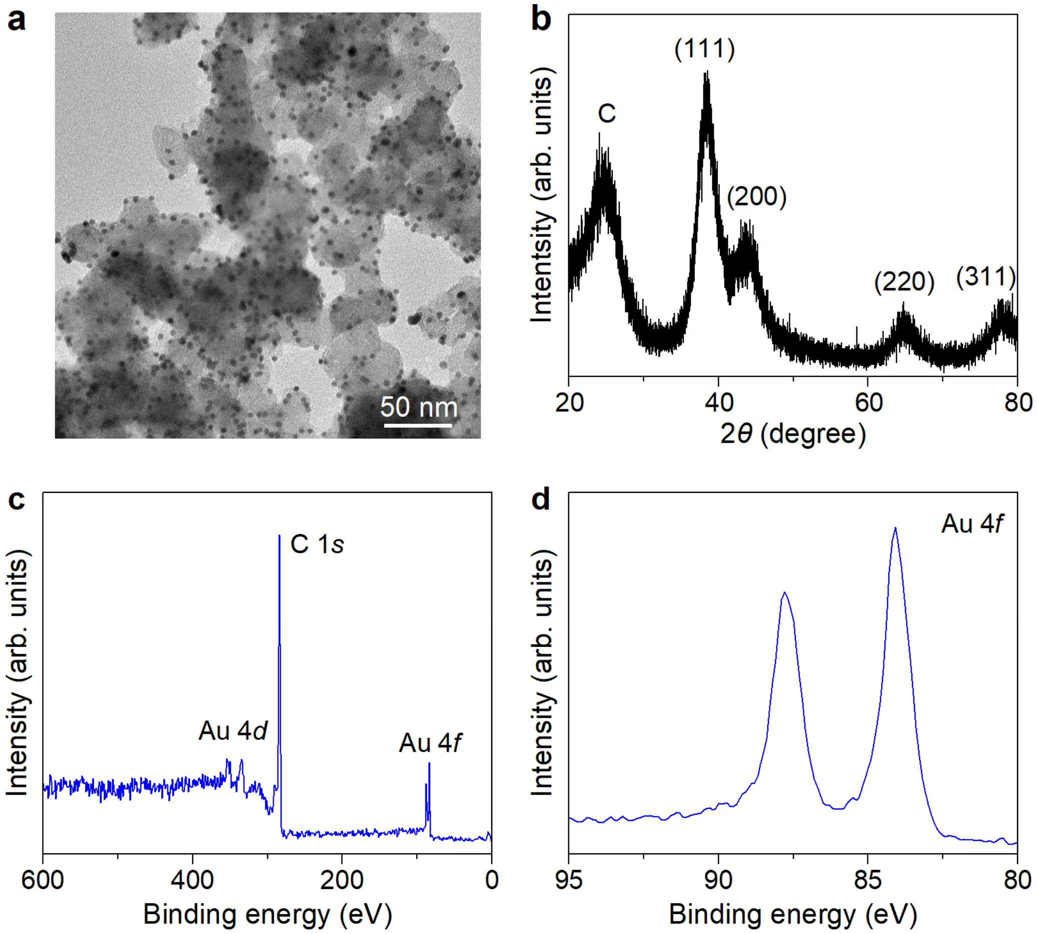

Supplementary Fig. 3. Characterization of the Au/C sample. **a,** TEM image. **b,** XRD pattern. **c,** XPS survey spectrum. **d,** High-resolution XPS spectrum of the Au 4*f* region. XPS spectra in **c** and **d** were adapted with permission from *J. Am. Chem. Soc.* **147**, 23277−23285 (2025). Copyright © 2025 American Chemical Society. Source data are provided as a Source Data file.

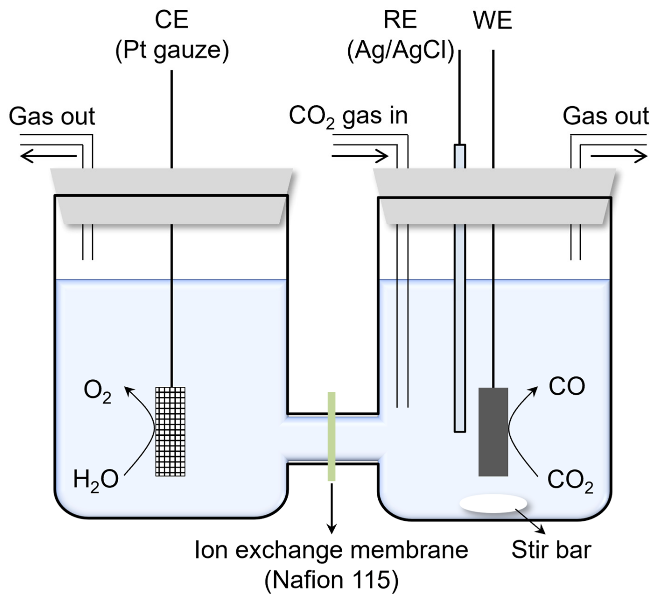

Supplementary Fig. 4. Schematic illustration of the H-cell setup used for CO_2RR studies.

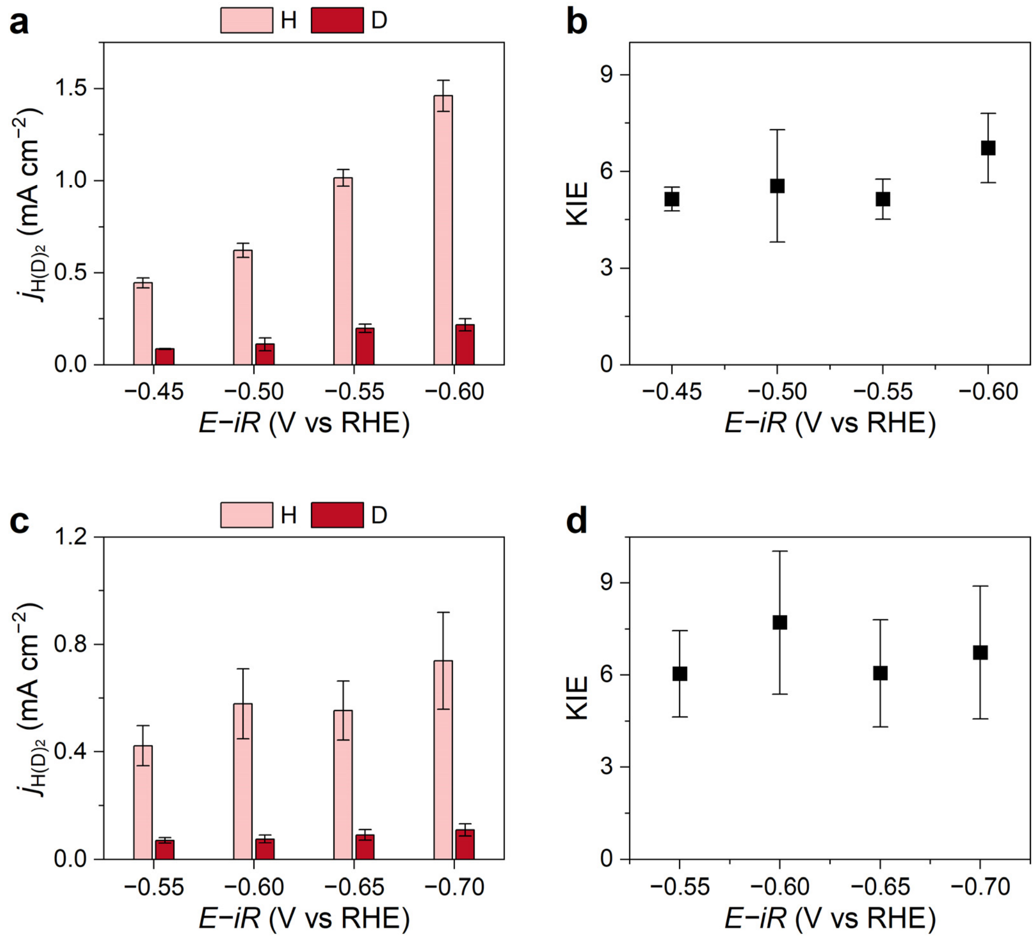

Supplementary Fig. 5. KIE analysis of concurrent HER on the Au/C and CoPc/CNTs electrodes. **a,c,** Partial current densities for H_2/D_2 production during CO_2RR on Au/C (**a**) and CoPc/CNTs (**c**) in 1 M $NaHCO_3$/H_2O and 1 M $NaDCO_3$/D_2O electrolytes. **b,d,** Corresponding KIE values on Au/C (**b**) and CoPc/CNTs (**d**). The applied potentials were 100% iR-compensated using the current-interrupt method ($NaHCO_3$/H_2O: R_u = 6.9 ± 0.3 Ω, pH = 7.4; $NaDCO_3$/D_2O: R_u = 8.1 ± 0.7 Ω, pD = 7.8; geometric electrode area = 0.5 cm^2). The error bars represent the standard deviation of three independent measurements. Source data are provided as a Source Data file.

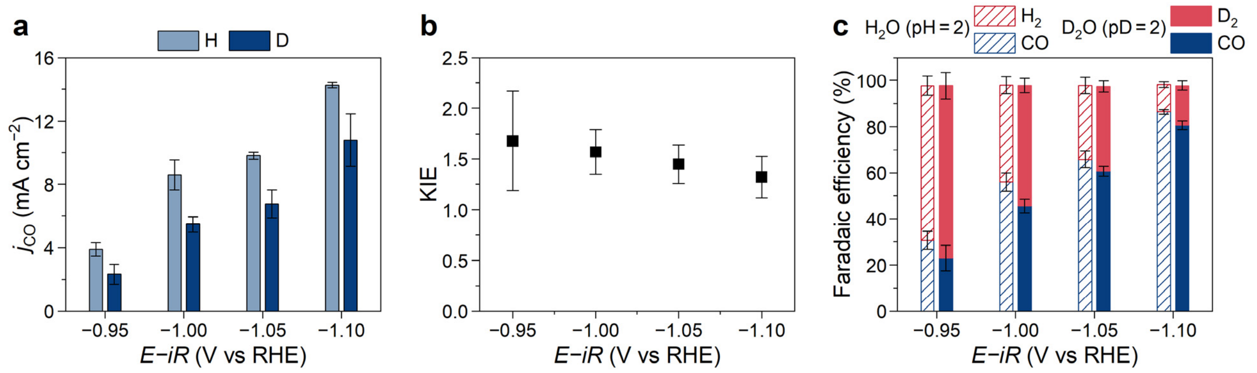

Supplementary Fig. 6. KIE analysis of CO_2RR on CoPc/CNTs under acidic conditions. **a,** Partial current densities for CO production, **b,** corresponding KIE values, and **c,** Faradaic efficiencies for CO_2RR on CoPc/CNTs in 1 M $NaClO_4/H_2O$ and 1 M $NaClO_4/D_2O$ electrolytes (pH/pD = 2). The applied potentials were 100% *iR*-compensated using the current-interrupt method ($NaClO_4/H_2O$: $R_u = 6.0 \pm 0.8\ \Omega$, pH = 2; $NaClO_4/D_2O$: $R_u = 7.0 \pm 0.6\ \Omega$, pD = 2; geometric electrode area = 0.5 cm^2). The error bars represent the standard deviation of three independent measurements. Source data are provided as a Source Data file.

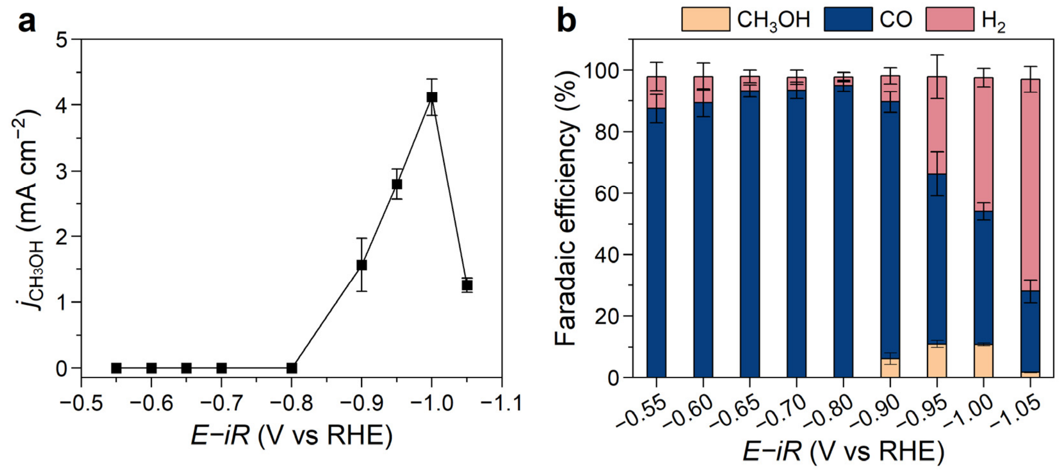

Supplementary Fig. 7. CO_2RR performance on CoPc/CNTs at more negative potentials. **a,** Partial current densities for CH_3OH production during CO_2RR on CoPc/CNTs in 1 M $NaHCO_3$ electrolyte. **b,** Corresponding Faradaic efficiencies. The applied potentials were 100% *iR*-compensated using the current-interrupt method ($R_u = 6.9 \pm 0.3\ \Omega$; pH = 7.4; geometric electrode area = 0.5 cm^2). The error bars represent the standard deviation of three independent measurements. Source data are provided as a Source Data file.

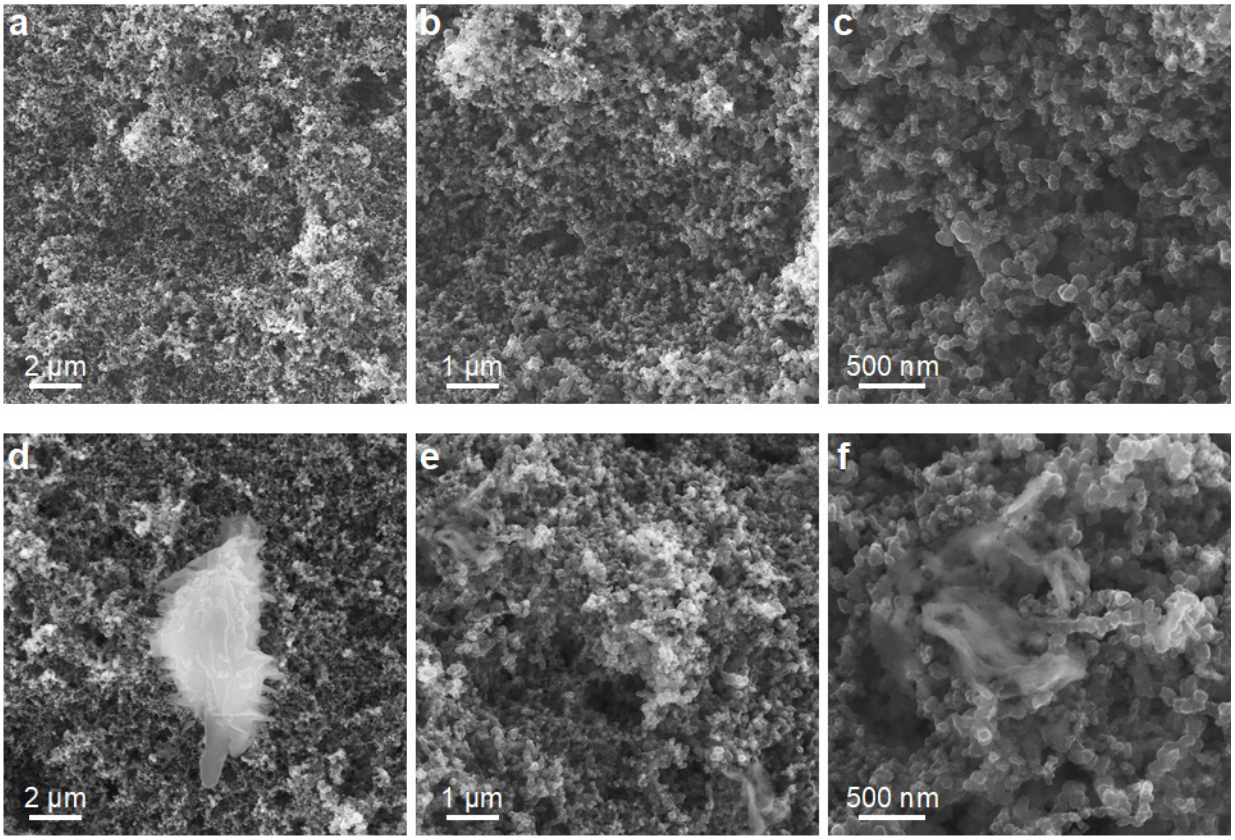

Supplementary Fig. 8. SEM images acquired at various magnifications of the AvCarb GDS2230 carbon substrate before (**a**–**c**) and after (**d**–**f**) the deposition of CoPc dissolved in DMF.

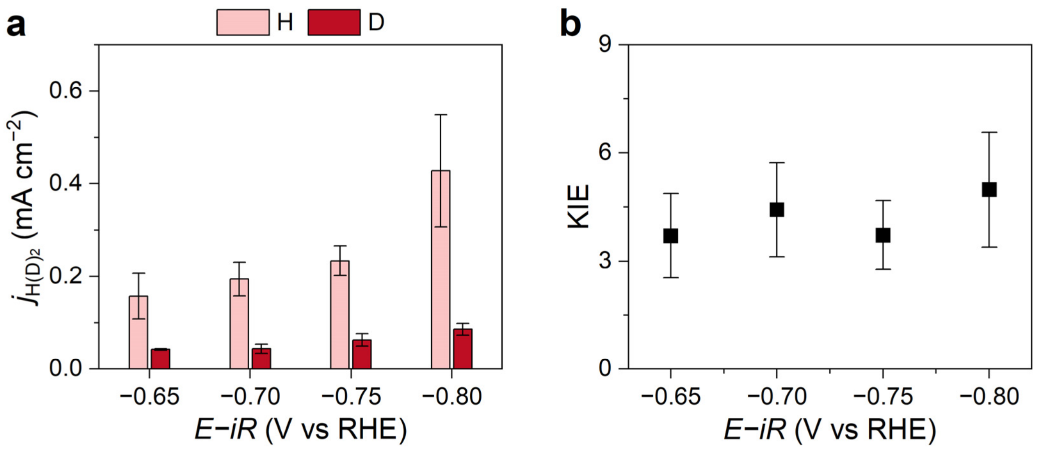

Supplementary Fig. 9. KIE analysis of concurrent HER on the aggregated CoPc electrode. **a,** Partial current densities for H_2/D_2 production during CO_2RR on the aggregated CoPc electrode in 1 M $NaHCO_3/H_2O$ and 1 M $NaDCO_3/D_2O$ electrolytes. **b,** Corresponding KIE values. The applied potentials were 100% *iR*-compensated using the current-interrupt method ($NaHCO_3/H_2O$: $R_u = 6.9 \pm 0.3\ \Omega$, pH = 7.4; $NaDCO_3/D_2O$: $R_u = 8.1 \pm 0.7\ \Omega$, pD = 7.8; geometric electrode area = 0.5 cm^2). The error bars represent the standard deviation of three independent measurements. Source data are provided as a Source Data file.

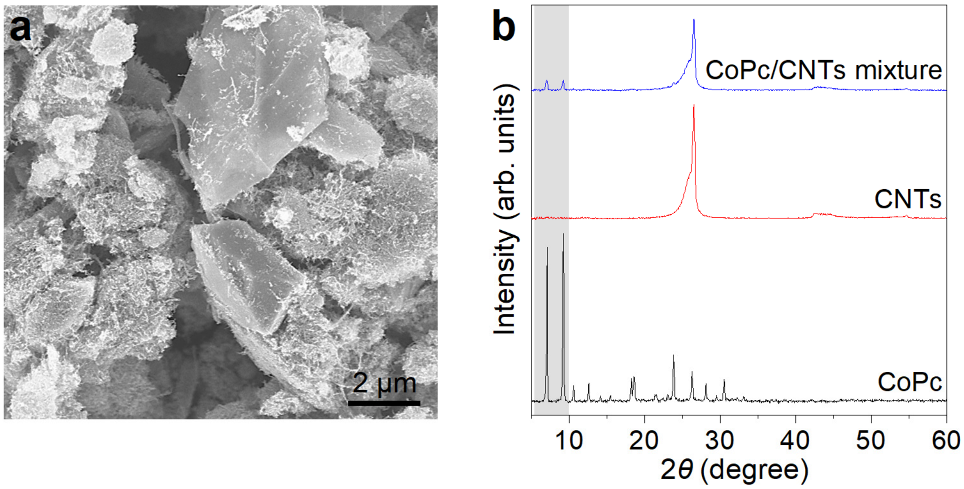

Supplementary Fig. 10. Characterization of the CoPc/CNTs mixture sample. **a,** SEM image of the CoPc/CNTs mixture sample. **b,** XRD pattern of the CoPc/CNTs mixture sample, with reference patterns of CNTs and CoPc for comparison. Source data are provided as a Source Data file.

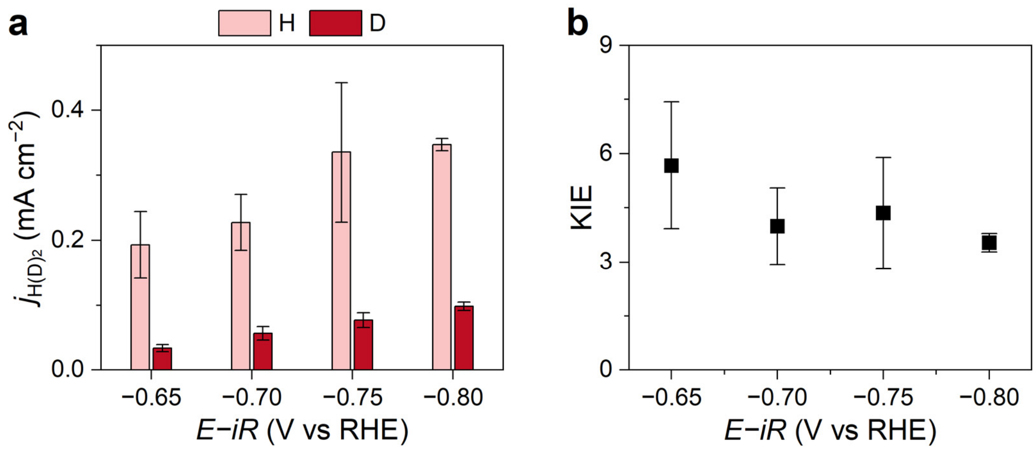

Supplementary Fig. 11. KIE analysis of concurrent HER on the CoPc/CNTs mixture electrode. **a,** Partial current densities for H_2/D_2 production during CO_2RR on the CoPc/CNTs mixture in 1 M $NaHCO_3/H_2O$ and 1 M $NaDCO_3/D_2O$ electrolytes. **b,** Corresponding KIE values. The applied potentials were 100% *iR*-compensated using the current-interrupt method ($NaHCO_3/H_2O$: $R_u = 6.9 \pm 0.3\ \Omega$, pH = 7.4; $NaDCO_3/D_2O$: $R_u = 8.1 \pm 0.7\ \Omega$, pD = 7.8; geometric electrode area = 0.5 cm^2). The error bars represent the standard deviation of three independent measurements. Source data are provided as a Source Data file.

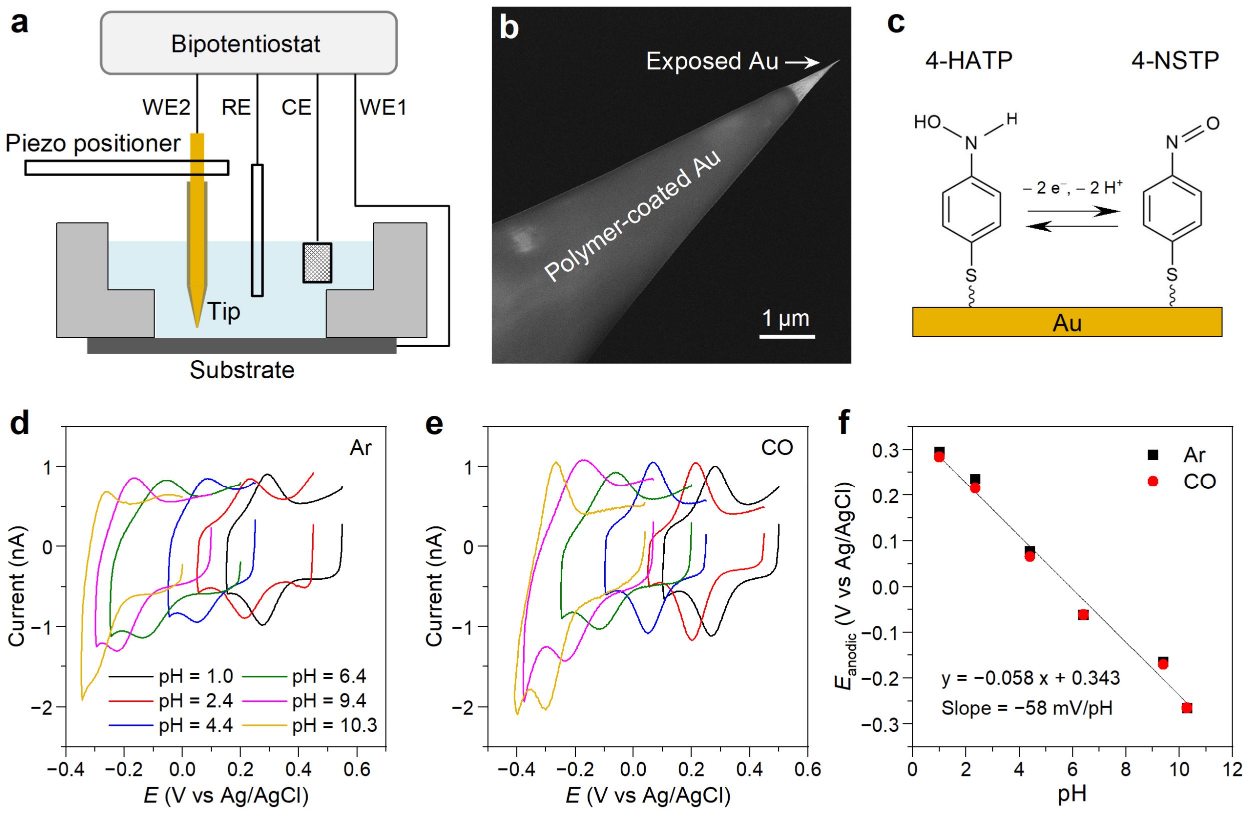

Supplementary Fig. 12. Probing local pH using scanning electrochemical microscopy (SECM). **a,** Schematic illustration of the customized SECM setup. **b,** SEM image of the Au nanoelectrode tip after electropolymerization coating, with exposed apex functionalized with 4-HATP/4-NSTP. **c,** Reversible 4-HATP/4-NSTP redox couple on Au used for pH sensing. **d–f,** Calibration curves correlating the mid-peak potential of the 4-HATP/4-NSTP anodic voltammetry with pH in Ar-saturated and CO-saturated electrolytes. The applied potentials in **d** and **e** were not *iR*-compensated. Source data are provided as a Source Data file.

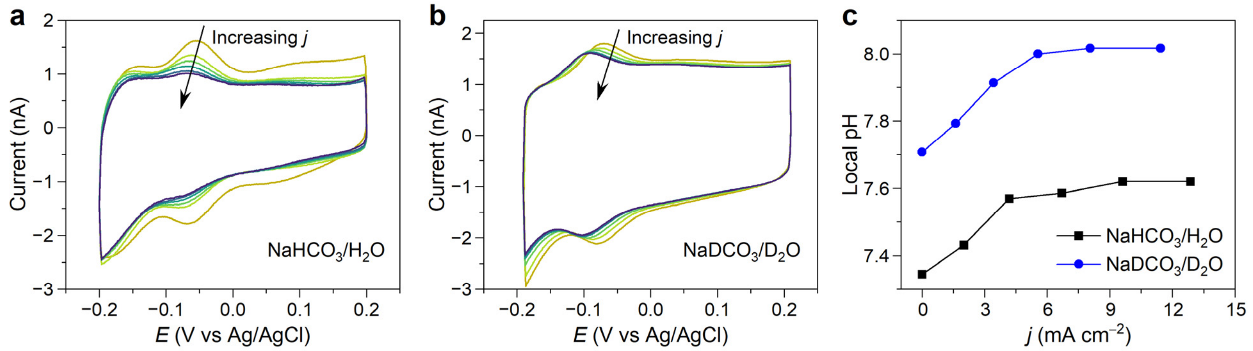

Supplementary Fig. 13. Operando probing of local pH during CO_2RR on CoPc/CNTs by SECM. **a,b,** Cyclic voltammograms recorded with a 4-HATP/4-NSTP functionalized Au nanoelectrode probe during CO_2RR at different current densities in 1 M $NaHCO_3/H_2O$ and 1 M $NaDCO_3/D_2O$ electrolytes. The applied potentials in **a** and **b** were not *iR*-compensated. **c,** SECM-measured local pH in the vicinity of the CoPc/CNTs electrode during CO_2RR at varying current densities. Source data are provided as a Source Data file.

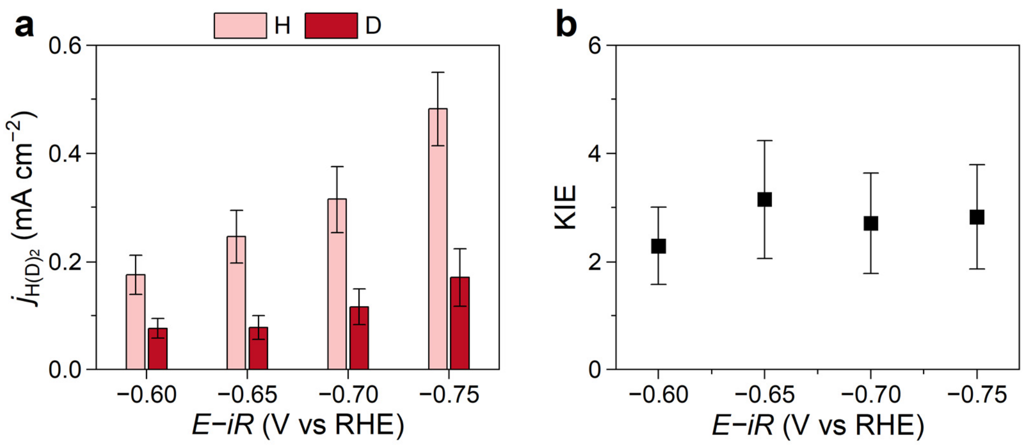

Supplementary Fig. 14. KIE analysis of concurrent HER on the CoPc/CNTs electrode in crown-ether-containing electrolytes. **a,** Partial current densities for H_2/D_2 production during CO_2RR on CoPc/CNTs in 1 M $NaHCO_3/H_2O$ + 18-crown-6 and 1 M $NaDCO_3/D_2O$ + 18-crown-6 electrolytes. **b,** Corresponding KIE values. The applied potentials were 100% *iR*-compensated using the current-interrupt method ($NaHCO_3/H_2O$ + 18-crown-6: R_u = 8.7 ± 0.3 Ω, pH = 7.4; $NaDCO_3/D_2O$ + 18-crown-6: R_u = 9.2 ± 0.3 Ω, pD = 7.8; geometric electrode area = 0.5 cm^2). The error bars represent the standard deviation of three independent measurements. Source data are provided as a Source Data file.

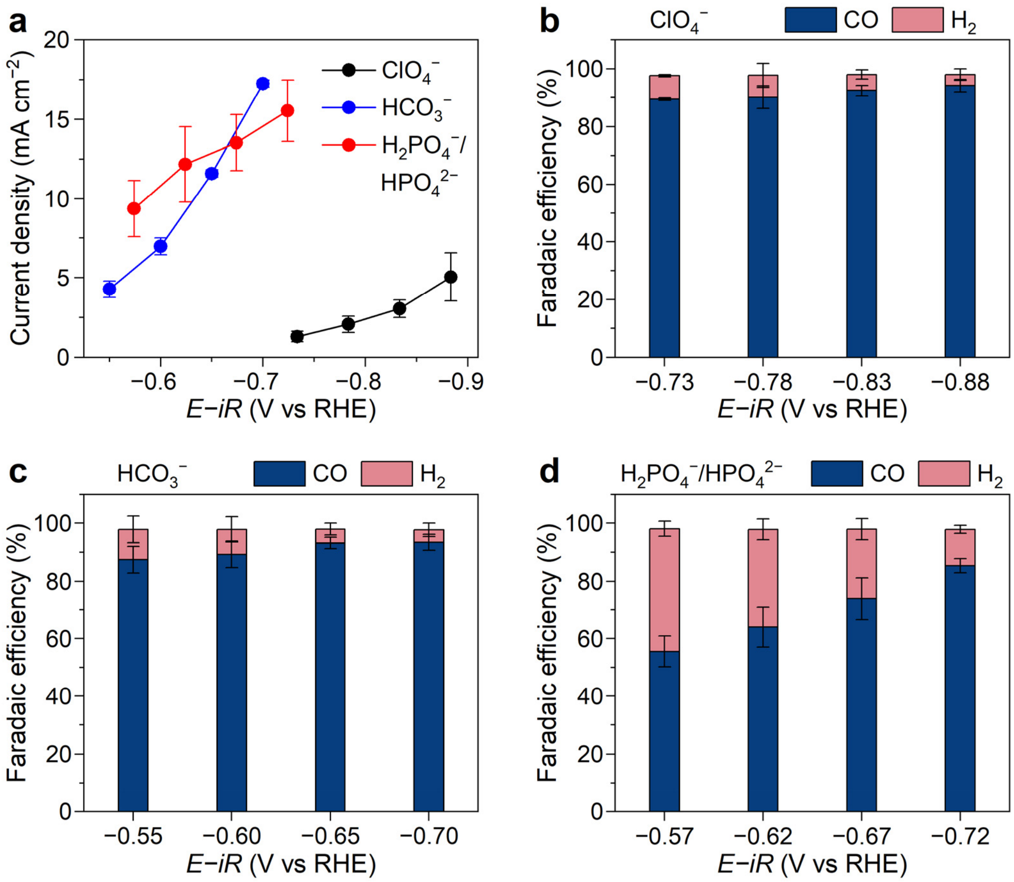

Supplementary Fig. 15. a, Total current densities, and **b–d,** Faradaic efficiencies for CO_2RR on CoPc/CNTs at selected potentials in electrolytes containing 1 M Na^+ paired with different anions: (**b**) ClO_4^-, (**c**) HCO_3^-, and (**d**) $H_2PO_4^-/HPO_4^{2-}$. The applied potentials were 100% *iR*-compensated using the current-interrupt method (ClO_4^-: $R_u = 7.2 \pm 0.1\ \Omega$, pH = 4.3; HCO_3^-: $R_u = 6.9 \pm 0.3\ \Omega$, pH = 7.4; $H_2PO_4^-/HPO_4^{2-}$: $R_u = 11.6 \pm 0.4\ \Omega$, pH = 6.6; geometric electrode area = 0.5 cm^2). The error bars represent the standard deviation of three independent measurements. Source data are provided as a Source Data file.

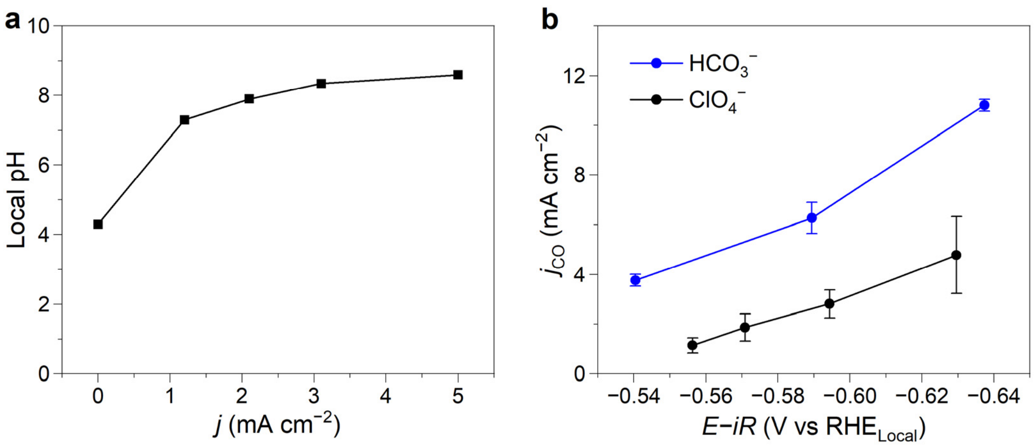

Supplementary Fig. 16. Effect of anion-dependent local pH on CO_2RR over CoPc/CNTs. **a,** Loal pH measured near the CoPc/CNTs electrode during CO_2RR at different current densities in 1 M $NaClO_4$ electrolyte using SECM. **b,** Replot of CO partial current densities during CO_2RR in the H-cell with 1 M $NaHCO_3$ or 1 M $NaClO_4$ at selected potentials, where the RHE potentials were calculated from SECM-measured local pH values. The error bars represent the standard deviation of three independent measurements. Source data are provided as a Source Data file.

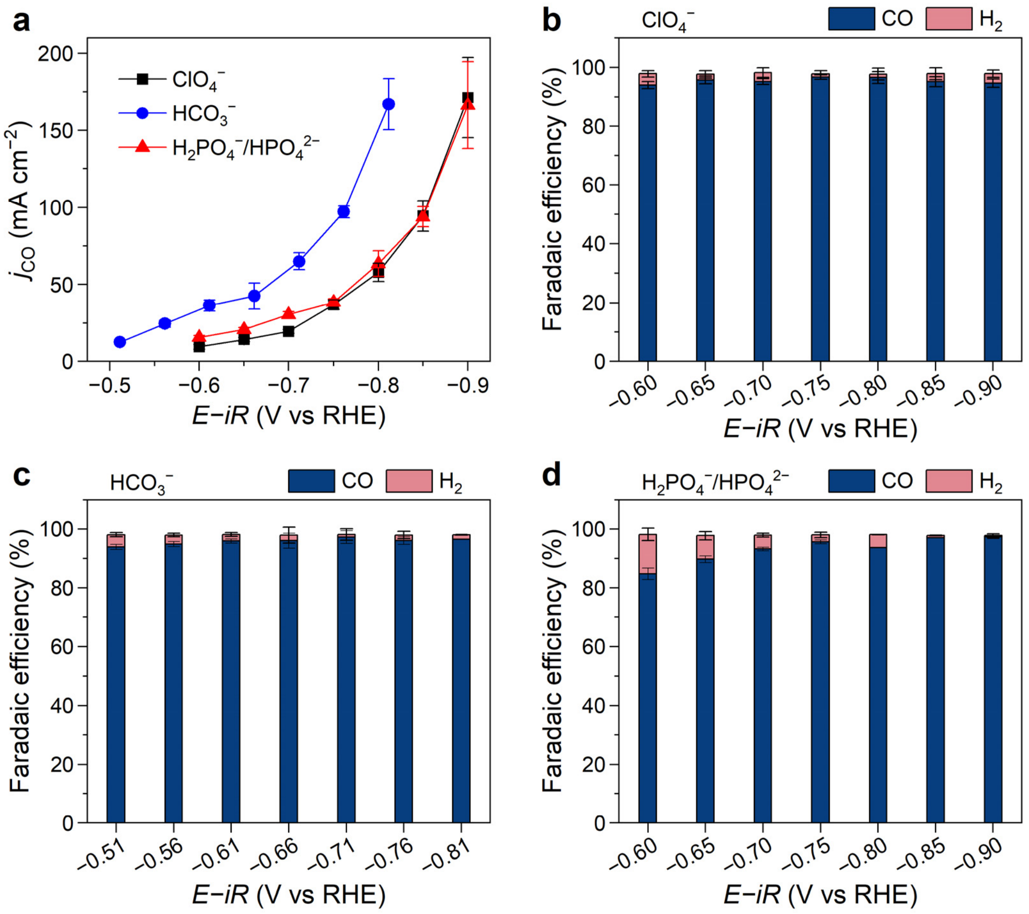

Supplementary Fig. 17. Effect of electrolyte anions on CO_2RR on CoPc/CNTs tested in a GDE flow cell. **a,** CO partial current densities, and **b–d,** corresponding Faradaic efficiencies for CO and H_2 production during CO_2RR at selected potentials in electrolytes containing 1 M Na^+ paired with different anions: (**b**) ClO_4^-, (**c**) HCO_3^-, and (**d**) $H_2PO_4^-/HPO_4^{2-}$. The applied potentials were 100% *iR*-compensated in the GDE flow cell using the current-interrupt method (ClO_4^-: $R_u = 4.9 \pm 0.8\ \Omega$, pH = 7.0; HCO_3^-: $R_u = 5.2 \pm 0.4\ \Omega$, pH = 8.5; $H_2PO_4^-/HPO_4^{2-}$: $R_u = 8.2 \pm 0.9\ \Omega$, pH = 7.0; geometric electrode area = 0.67 cm^2). The error bars represent the standard deviation of three independent measurements. Source data are provided as a Source Data file.
